# Palatability of Protein Hydrolysates from Industrial Byproducts for Nile Tilapia Juveniles

**DOI:** 10.3390/ani9060311

**Published:** 2019-05-31

**Authors:** Denis Rogério Sanches Alves, Suzana Raquel de Oliveira, Thiago Gabriel Luczinski, Isabela Guterres Pinto Paulo, Wilson Rogério Boscolo, Fábio Bittencourt, Altevir Signor

**Affiliations:** 1 Centre of Engineering and Exact Sciences, State University of West Paraná–UNIOESTE, Toledo 520, Paraná, Brazil; suzanarachel@hotmail.com (S.R.d.O.); thiago_l@live.com (T.G.L.); isabela.guterres@gmail.com (I.G.P.P.); wilsonboscolo@hotmail.com (W.R.B.); fabio.gemaq@gmail.com (F.B.); altevir.signor@gmail.com (A.S.); 2 Department of Engineering and Exact Sciences, Federal University of Paraná- UFPR (Sector Palotina), Palotina 2153, Paraná, Brazil

**Keywords:** feed attractant, wastes, aquaculture, nutrition, behavior, free amino acids

## Abstract

**Simple Summary:**

Aquaculture is highlighted as one of the most important activities to generate food with a sustainable character. Developing the production chain as a whole is considerably challenging, especially aiming to sustain its constant growth, thus studies regarding alternative feeds are required in order to meet the nutritional demands of this species. The protein ingredients are responsible for the larger share of this cost at 40–70% of the total expenses, and among protein sources, fishmeal is the most used in fish feeds. Improving the use of these byproducts (e.g., from poultry and swine productions) by adopting optimizing technologies represents an environmental, economic, and social alternative. In this sense, this study aimed at determining the palatability of protein hydrolysates made of swine liver, swine mucosa, poultry byproducts, and feathers for Nile tilapia juveniles. Protein hydrolysates increased the final consumption of pellets in comparison to fishmeal and provided the highest palatability index without altering the feeding behavior. Protein hydrolysates from byproducts from the industry of poultry and swine can be used in a sustainable way in diets for Nile tilapia juveniles in replacement of fishmeal.

**Abstract:**

This study was performed with the aim of determining the palatability of diets containing poultry and swine protein hydrolysates for Nile tilapia. Five experimental diets were made with a 5% inclusion level of fishmeal (FPE), poultry protein hydrolysate (PHF), liquid swine hydrolysate (PHS), feather protein hydrolysate (PHP), and swine mucosa hydrolysate (PHM). Five juveniles (2.81 ± 0.17 g) were placed in 10 L aquariums and fed five times a day after a raffle of offered diets. In each feeding throughout the day, 30 pellets were offered per fish. The experimental design was completely randomized, with five replicates per day. A three-minute footage period was established for each feeding with a digital camera. The following feeding behaviors were observed: time to capture the first pellet, number of pellet rejections, number of approaches without capturing the pellet, and number of consumed pellets. No observed parameters presented significant differences (*p* > 0.05). PHF provided a palatability index of 12.27%, while PHM had an index of 9.33%, PHF of 8.77%, and PHP of 7.74%. Both diets PHS and PHM increased the final consumption of pellets in comparison to FPE by more than 10%, despite the absence of a significant effect.

## 1. Introduction

According to the most recent report [1], human societies are facing an enormous challenge to supply food for a population of above 9 billion people, and to do so, aquaculture is highlighted as one of the most important activities to generate food with a sustainable character.

Brazil has a great potential for aquaculture production mainly due to the abundance of marine and freshwater environments, composing approximately 5.3 million hectares of freshwater in natural and artificial reservoirs [2]. Allied to this fact, the country has an enormous variety of species with rearing potential.

Although more than 70 species of tilapia are known, the Nile tilapia (*Oreochromis niloticus*) stands out by its great rearing potential, easy reproduction, and adaptability to handlings, besides its acceptance of several feeding sources and the high quality of the meat, which is greatly accepted by the final consumer [3,4,5]. This species is the fourth most produced in the world and the most produced species in Brazil [1,6]. Nile tilapia is a phytoplanktophage species of low trophic level in the food chain, thus contributing to the reduction of aquatic eutrophication. Therefore, it is not an invasive species in Brazil.

With an increasing aquaculture production, studies regarding alternative feeds are required in order to meet the nutritional demands of this fish species [7,8]. Feeds represent the most expensive operational cost in aquaculture, with protein ingredients being responsible for the larger share of this cost at 40–70% of the total expenses [9,10,11]. Among protein sources, fishmeal is the most used in fish feeds. However, due to the high demand for this ingredient, its supply is expected to reduce or be limited in the future [12].

This situation led the aquaculture industry to adapt and search for alternative food sources that could be included in fish diets as protein ingredients with high palatability, also in order to reduce waste generation [13,14]. One way to reduce costs with commercial feeds is using agro-industrial byproducts, since such wastes are currently underused or disposed of inadequately, causing environmental damage [8]. Therefore, improving the use of these byproducts (e.g., from poultry and swine production) by adopting optimizing technologies represents an environmental, economic, and social alternative [15]. Factors such as high supply, good biological quality, and low cost of these materials raise the interest for employment in animal nutrition, especially as a way of facilitating processes and improving the nutritional quality of these products [12,15,16].

Brazil has well-established production of swine and poultry, which generates large volumes of wastes and nonedible byproducts such as viscera, feathers, and guts. In this context, the byproducts could be used as raw materials for the production of feed ingredients such as meals, oils, and protein hydrolysates at a low cost [8,12,16,17].

A protein hydrolysate is a result of the solubilization of proteins, and can be obtained either by chemical hydrolysis (acid or alkaline) or enzymatic hydrolysis with the addition of plant, animal, or microbial enzymes to the material [8,18,19]. The hydrolysis process is a chemical reaction that modifies the functional properties of feeds and promotes the break of protein chains into amino acids and multiple-sized peptides with high protein content which are more easily absorbed by the animals [16,19,20]. The enzymatic hydrolysis is more advantageous to promote the protein hydrolysis, seeing that it adds value to the nutritional and functional characteristics of the raw materials, besides being a controlled rapid and practical process that causes low environmental impact when compared to acid and alkaline hydrolysis [16,21,22].

Studies regarding digestibility, performance, and influence on growth are common when new diets are formulated [11]. However, one of the most important data is commonly neglected in such studies: the acceptance of the ingredient by the animal. A feed will only be ideal if besides its high nutritional quality, it presents organoleptic characteristics that facilitate the detection and stimulates the ingestion of the diet [23]. Therefore, studies regarding the palatability of new diets should precede the commonly performed studies.

In this sense, this study aimed at determining the palatability of protein hydrolysates made of swine liver, swine mucosa, poultry byproducts, and feathers for Nile tilapia juveniles. We tested experimental diets with higher protein content and hydrolysates with higher free amino-acid content than our previous study [24]. Use of these newly prepared diets gave higher palatability indices and appointed a better way to achieve sustainable aquaculture.

## 2. Materials and Methods

The experiment was conducted in the Laboratory of Aquaculture of the Aquaculture Management Study Group-GEMAq, University of West Paraná (UNIOESTE), campus Toledo/PR, Brazil, between May and June 2018. The procedures presented in this study were approved by the Ethics Committee on Animal Use (CEUA) of the State University of West Paraná, according approval to an Experimental Certificate of Animal Use (nº 11/18).

The powder protein hydrolysates used were supplied by the company Brasil Foods S/A (sector BRF^®^ Ingredients) (Concórdia-SC, Brazil). The hydrolysates were obtained enzymatically, and their hydrolysis degree (percentage of cleaved peptide bonds) was determined by the orthoftaldehyde method [25] (as you can see in Supplementary Materials—Figure S1: The OPA-reaction and Table S1: Value of constants a, b, and h_tot_ for different protein raw materials). We obtained protein hydrolysis degrees of 36% for swine mucus, 27% for swine liver, 21% for poultry, and 15% for feather.

Five experimental diets were formulated, according to the nutritional recommendations [10]. The experimental diets were:

(a) FPE (positive control) = diet with 5% inclusion of fishmeal;
(b) PHF = diet with 5% inclusion of poultry protein hydrolysate;
(c) PHP = diet with 5% inclusion of feather protein hydrolysate;
(d) PHM = diet with 5% inclusion of swine mucus protein hydrolysate;
(e) PHS = diet with 5% inclusion of swine liver protein hydrolysate (Table 1 and Table 2).

The protein hydrolysates were submitted to crude fiber, fat, dry matter, and energy analysis (Table 3). The analyses were performed according to the methodology described by Instituto Adolfo Lutz [26], with the exception of crude energy, which was determined with the aid of a calorimetric pump. All analyses were performed at the Food Quality Laboratory (LQA) of the GEMAq.

All ingredients were initially milled and sieved (0.3 mm) in a hammer mill (model MCs 280, Vieira Moinhos e Martelo, Tatuí-SP, Brazil). In order to extrude the diets, the blends were moistened with 20% water and homogenized, followed by a 15-minute mixing period in a mechanic mixer type “Y” (model MA 200, Marconi Equipamentos Laboratoriais, Piracicaba-SP, Brazil). The diets were then extruded using a 1.0 mm diameter sieve in an equipment model Ex-Micro with a capacity of 10 kg h^−1^ (Exteec Máquinas, Ribeirão Preto-SP, Brazil). After the extrusion, the diets were placed in an air-ventilated oven for 24 h at 55 °C (model TE-394/3-D, Tecnal Equipamentos Científicos para Laboratórios, Piracicaba-SP, Brazil).

The amino acid composition of the hydrolyzed proteins (Table 4) was assessed by means of the method MA-009 [27,28] in the commercial laboratory (CBO Análises Laboratoriais Ltda., Valinhos-SP, Brazil).

Five Nile tilapia juveniles (2.81 ± 0.17 g) were distributed individually in five aquariums with 10 L of useful volume, which were coated with latex waterproof material and individually equipped with aeration systems and temperature control, with 15 W thermostats. Each aquarium also had a central hole for pellets deposition.

Water quality parameters were monitored in three steps (beginning, middle, and end of the experimental period). Water temperature, pH, and dissolved oxygen were measured with the aid of a multiparameter probe. The photoperiod was natural and both physical and chemical characteristics of the water were similar between aquariums, with a mean temperature of 27.8 ± 0.35 °C, pH 7.53 ± 0.04, total ammonia 0.3 ± 0.13 ppm, toxic ammonia 0.01 ± 0.005 ppm, and dissolved oxygen 5.54 ± 0.13 mg L^−1^. All parameters were considered adequate according to those recommended for the species’ development [29].

A foam barrier was placed on the aquariums’ surroundings in order to isolate them from the routine activities of the laboratory, thus minimizing possible effects on the animals’ behavior. The fish underwent a 10-day adaptation and training period so they could get used to human presence through the recording of feed behavior during the feeding assay and during the quantification of the pellets needed to reach apparent satiety. During this period, fish were fed with a commercial diet (40% crude protein, 1 mm pellet) and after this acclimatization, the palatability assay started.

Fish were fed five times a day at 08:00 h, 10:30 h, 13:00 h, 15:30 h, and 18:00 h. On a daily basis, all aquariums were siphoned for waste removal and a water renewal of approximately 10%. In each feeding throughout the day, 30 pellets were offered per fish. The experimental design was completely randomized, with five replicates per day. A draw of the treatment to be offered per fish was performed every day. A three-minute footage period was established for each feeding with a digital camera, at the exact moment the diet was introduced in the aquarium. The assay lasted for 12 days; thus, 300 pieces of footage were obtained (5 fish × 5 feedings × 25 assays per day) and for each tested treatment, 60 trials were made.

The palatability assay was performed according to the methodology described by Kasumyan et al. [30,31,32]. These authors proposed the palatability index as a quantitative estimate of the gustative preference (in percentage), using the formula:

(1) IP=((R−C)÷(R+C))×100

in which: IP is the palatability index; R is the consumption of pellets of the test diet; and C is the consumption of pellets of the control diet.

After data collection, the footages were examined in order to analyze the following feeding behaviors [23]: (a) time to capture the first pellet (seconds); (b) number of pellet rejection after capture; (c) number of approaches without capturing the pellet; (d) number of consumed pellets. The palatability index was then calculated for each treatment.

All data were submitted to a parametric variance analysis (ANOVA), and in case of significant effect (*p* < 0.05), the multiple comparison test of Tukey was performed at a 5% significance level. Before the analyses, the normality of data was verified by the Shapiro–Wilk’s test, as well as the homogeneity of variances (Levene’s test). The analyses were performed with the aid of the software Statistic (Version 7.1; TIBCO® Software Inc., Palo Alto, CA, USA).

## 3. Results

All diets presented a positive palatability index in comparison to FPE. The diet PHS provided an index of 12.27%, PHM of 9.33%, PHF of 8.77%, and PHP an index of 7.74% (Table 5). Regarding the data analysis of palatability, none of the evaluated parameters presented significant differences (*p* > 0.05).

All diets provided a higher consumption of pellets in comparison to FPE. It is noteworthy that both PHS and PHM increased the final consumption of pellets in comparison to FPE in more than 10%, despite the absence of a statistically significant index.

Although no statistical differences (*p* > 0.05) were found for the number of rejections after capturing the pellet, it is noteworthy that all diets presented a lower numeric value in relation to FPE. The lowest mean value of rejections was observed in PHP, with 0.34 pellets, followed by the diet PHF (0.39 pellets), PHM (0.47 pellets), and PHS with 0.50 pellets. The diet PHP showed about 42% lower rejection after capturing in relation to FPE.

## 4. Discussion

The chemical composition presented by the protein hydrolysates (Table 3) was similar to other hydrolysates of animal origin mentioned in the literature, such as: feather hydrolysate, 83.9% and 77% of crude protein, 7.2% and 3% of lipids [33,34]; swine liver protein hydrolysate, 72% of crude protein and 14% of lipids [35]; tilapia hydrolysate (6529 kcal kg^−1^ of crude energy) [12]; sardine hydrolysate (with both low and high degree of hydrolysis), with 73.4% and 82.5% of crude protein, 6.4% and 2.4% of lipids, respectively [36]. Both the nature and quality of the raw materials are factors that may influence the obtained results and affect the characteristics of the diets, as well as the quality and functionality of the final product [15,37].

Despite the absence of a significant effect, the highest values found for the palatability index, pellets consumption, number of rejections after capture, approaches without capturing the pellet, and time to capture the first pellet in all diets containing poultry byproducts might be related to the amino acid content of such products [38] (Table 4). Amino acids are responsible for chemical signals received by the gustatory system of fish, classified as stimulants, and may differentiate the attractiveness of a feed, such as cysteine, betaine, glutamic acid, serine, glycine, alanine, proline, methionine, cysteine, phenylalanine, arginine, tyrosine, valine, leucine, and glutamine [23,30,31,33,39,40,41,42,43].

The presence of free amino acids and peptides of low molecular weight is considered to increase a feed’s attractiveness [31,41,43,44,45]. Replacing fishmeal with protein ingredients of animal origin with a supplementation of lysine and methionine indicated a positive correlation associated to the improvement of the palatability for gibel carp (*Carassius auratus gibelio*) [46]. When studying the inclusion of protein hydrolysates of poultry and swine in replacement of fishmeal for Nile tilapia juveniles, the present study gave improved palatability indices in comparison to our previous study [24] (12.27%, 7.74% vs. 10.82%, −6.66%), which might be induced by higher crude protein content in the current experimental diets (40% vs. 30%) and higher free amino-acid content in the hydrolysates (30.06%, 4.81% vs. 15.36%, 1.33%). Use of swine mucus hydrolysate newly gave a good result with a positive palatability index.

No significant difference was verified for the feeding behavior regarding the time to capture the first pellet (Table 5). However, it was observed that for all diets containing protein hydrolysates, the Nile tilapia displayed a rapid pellet consumption, with an average of 1.12 seconds, which may be explained by the amount of free amino acids detected in such diets (Table 4). When studying the use of protein hydrolysates derived from meat products in diets [47], goldfish (*Carassius auratus*) presented high palatability among diets containing such hydrolysates. Similarly, our previous study [24] observed that Nile tilapia juveniles fed with poultry, feather, and swine liver hydrolysates in replacement of fishmeal showed an average time of 0.82 seconds to capture feed, and highlighted that the positive differences found for the palatability index, pellet consumption, and number of rejections after capture for the poultry protein hydrolysate may be associated to the concentration of free amino acids. The rapid capture of pellets is an important factor in aquaculture, seeing that it reflects a lower nutrient loss to the water, resulting in the ingestion of a balanced feed without causing great impact in the environment [48].

In this study, it is noteworthy that all diets containing protein hydrolysates presented a lower number of approximations without capturing the pellet in comparison to FPE, despite the absence of significance. Juveniles of the catfish (*Rhamdia quelen)* fed with sardine waste hydrolysate, in comparison to treatments containing extract of Nile tilapia muscle (positive control) and distilled water (negative control), would swim faster in the experimental tanks trying to find the feed, thus the chemical stimulus favored the search and ingestion of the diet [49].

It can be observed that diets containing protein hydrolysates showed a trend of better consumption. Evaluating the addition of powder hydrolysates derived from both poultry and swine industries may provide valuable information about the chemosensory behavior of fish, besides improving the organoleptic characteristics and palatability of diets, seeing that some substances improve the attractiveness and palatability, thus increasing feed consumption [50,51,52,53]. Additionally, this study can generate information that may be applied in the feed industry, such as the utilization of byproducts that would be discarded, leading to environmental damage [8].

## 5. Conclusions

Protein hydrolysates from byproducts from the industry of poultry and swine can be used in a sustainable way in diets for Nile tilapia (*Oreochromis niloticus*) juveniles in replacement of fishmeal without altering the palatability index and the feeding behavior.

## Figures and Tables

**Table 1:** Composition of the experimental diets used in the evaluation of the palatability for Nile tilapia (*Oreochromis niloticus*), based on dry matter.

Ingredients	Diets				
	FPE	PHF	PHP	PHM	PHS
Soy bran (45%) ^1^	36.15	33.94	32.59	36.24	34.36
Cornmeal	20.00	20.00	20.00	20.00	20.00
Rice grits (8.5%) ^1^	6.64	7.74	8.54	5.00	7.41
Poultry guts meal	10.00	10.00	10.00	10.00	10.00
Feather meal	8.00	8.00	8.00	8.00	8.00
Fishmeal (55%) ^1^	5.00	0.00	0.00	0.00	0.00
Poultry protein hydrolysate	0.00	5.00	0.00	0.00	0.00
Feather protein hydrolysate	0.00	0.00	5.00	0.00	0.00
Swine mucus protein hydrolysate	0.00	0.00	0.00	5.00	0.00
Swine liver protein hydrolysate	0.00	0.00	0.00	0.00	5.00
Corn gluten meal (60%) ^1^	5.00	5.00	5.00	5.10	5.00
Blood meal	2.00	2.00	2.00	2.00	2.00
Soy oil	2.09	2.41	2.53	2.67	2.21
Dicalcium phosphate	1.39	2.00	2.12	1.94	2.08
Mineral–vitamin supplement ^2^	0.60	0.60	0.60	0.60	0.60
Calcitic lime	0.31	0.56	0.49	0.69	0.51
L-lysine HCL	0.80	0.71	1.02	0.72	0.79
L-threonine	0.63	0.62	0.64	0.62	0.64
Common salt	0.50	0.50	0.50	0.50	0.50
DL-methionine	0.42	0.42	0.47	0.43	0.41
Vitamin C (35%)	0.20	0.20	0.20	0.20	0.20
Choline chloride (60%)	0.15	0.15	0.15	0.15	0.15
Antifungal (calcium propionate)	0.10	0.10	0.10	0.10	0.10
Antioxidant (BHT) ^3^	0.02	0.02	0.02	0.02	0.02
Total	100.00	100.00	100.00	100.00	100.00

FPE (positive control) = diet with inclusion of fishmeal; PHF = diet with inclusion of poultry protein hydrolysate; PHP = diet with inclusion of feather protein hydrolysate; PHM = diet with inclusion of swine mucus protein hydrolysate; PHS = diet with inclusion of swine liver protein hydrolysate; ^1^ Crude Protein Content; ^2^ Levels of guarantee by kilogram of the product: vit. A, 500,000 UI; vit. D_3_, 200,000 UI; vit. E, 5000 mg; vit. K_3_, 1000 mg; vit. B_1_, 1500 mg; vit. B_2_, 1500 mg; vit. B_6_, 1500 mg; vit. B_12_, 4000 mg; folic acid, 500 mg; calcium pantothenate, 4000 mg; vit. C, 15,000 mg; biotin, 50 mg; inositol, 10,000 mg; nicotinamide, 7000 mg; choline, 40,000 mg; cobalt, 10 mg; copper, 500 mg; iron, 5000 mg; iodine, 50 mg; manganese, 1500 mg; selenium, 10 mg; zinc, 5000 mg; ^3^ Butylhydroxytoluene.

**Table 2:** Calculated composition of the experimental diets used in the evaluation of palatability for Nile tilapia (*Oreochromis niloticus*).

Calculated Composition	Diets				
	FPE	PHF	PHP	PHM	PHS
Starch (%)	22.73	23.29	23.72	21.55	23.09
Total arginine (%)	2.50	2.50	2.49	2.43	2.45
Calcium (%)	1.31	1.31	1.31	1.30	1.31
Digestible energy (kcal kg^−1^)	3.323	3.379	3.323	3.150	3.371
Total phenylalanine (%)	1.92	1.90	1.89	1.93	1.91
Crude fiber (%)	2.35	2.24	2.17	2.35	2.26
Available phosphorus (%)	0.85	0.81	0.79	0.76	0.79
Total phosphorus (%)	1.00	1.00	1.00	1.00	1.00
Fat (%)	6.00	6.00	6.00	6.00	6.00
Total histidine (%)	0.90	0.91	0.80	0.92	0.90
Total isoleucine (%)	1.64	1.63	1.62	1.64	1.63
Total leucine (%)	3.34	3.31	3.30	3.34	3.33
Total lysine (%)	2.60	2.60	2.60	2.60	2.60
Total methionine (%)	1.00	1.00	1.00	1.00	1.00
Crude protein (%)	40.00	40.00	40.00	40.00	40.00
Fish digestible protein (%)	34.98	35.21	31.5	32.57	35.21
Total threonine (%)	2.20	2.20	2.20	2.20	2.20
Total tryptophan (%)	0.41	0.42	0.38	0.40	0.42
Total valine (%)	2.08	2.07	2.09	2.08	2.09

FPE (positive control) = diet with inclusion of fishmeal; PHF = diet with inclusion of poultry protein hydrolysate; PHP = diet with inclusion of feather protein hydrolysate; PHM = diet with inclusion of swine mucus protein hydrolysate; PHS = diet with inclusion of swine liver protein hydrolysate.

**Table 3:** Chemical composition of the protein hydrolysates used in the evaluation of palatability for Nile tilapia (*Oreochromis niloticus*), based on dry matter.

Parameters	Poultry Protein Hydrolysate	Feather Protein Hydrolysate	Swine Mucus Protein Hydrolysate	Swine Liver Protein Hydrolysate
Crude protein (%)	78.18	76.33	59.28	78.04
Lipids (%)	8.13	2.61	0.00	11.97
Dry matter (%)	93.57	96.27	94.01	93.62
Crude energy (Kcal kg^−1^)	5900	4900	3320	5320

**Table 4:** Profile of free amino acids of the hydrolyzed proteins used in the evaluation of palatability for Nile tilapia (*Oreochromis niloticus*), based on dry matter.

Chemical Composition	Swine Mucus Protein Hydrolysate (%)	Poultry Protein Hydrolysate (%)	Swine Liver Protein Hydrolysate (%)	Feather Protein Hydrolysate (%)
Aspartic Acid	2.31	0.59	0.25	0.12
Glutamic Acid	2.93	1.41	0.72	0.26
Serine	1.67	0.48	0.64	0.26
Glycine	1.61	0.41	0.42	0.16
Histidine	0.93	0.33	0.31	0.16
Taurine	0.22	0.48	0.12	0.07
Arginine	1.28	1.32	0.40	0.13
Threonine	1.61	0.55	0.57	0.14
Alanine	2.30	0.93	1.14	0.31
Proline	1.64	0.41	0.58	0.12
Tyrosine	1.51	0.82	0.73	0.52
Valine	2.11	0.82	1.08	0.33
Methionine	0.84	0.47	0.41	0.38
Cystine	0.20	0.16	0.14	0.27
Isoleucine	1.44	0.56	0.81	0.38
Leucine	3.12	1.47	2.09	0.47
Phenylalanine	1.54	0.78	0.86	0.43
Lysine	2.77	1.14	0.90	0.29
Asparagine	0.03	0.03	0.08	None detected
Total	30.06	13.17	12.25	4.81

**Table 5:** Mean values of the palatability test of different protein hydrolysates for Nile tilapia (*Oreochromis niloticus*), in comparison to fishmeal (positive control).

Treatments	Palatability Index (%)	Consumption of Pellets (%)	Number of Rejections after Capturing the Pellet	Number of Approaches without Capturing the Pellet	Time to Capture the First Pellet (Seconds)
FPE	0	74.72 ± 28.75	0.59 ± 0.87	0.90 ± 0.51	1.49 ± 0.34
PHS	12.27	83.44 ± 16.05	0.50 ± 0.92	0.72 ± 0.39	1.23 ± 0.31
PHM	9.33	83.39 ± 9.99	0.47 ± 0.57	0.79 ± 0.60	1.13 ± 0.21
PHF	8.77	82.26 ± 11.43	0.39 ± 0.36	0.81 ± 0.56	1.14 ± 0.23
PHP	7.74	81.99 ± 14.89	0.34 ± 0.59	0.59 ± 0.62	1.01 ± 0.06

FPE (positive control) = diet with inclusion of fishmeal; PHF = diet with inclusion of poultry protein hydrolysate; PHP = diet with inclusion of feather protein hydrolysate; PHM = diet with inclusion of swine mucus protein hydrolysate; PHS = diet with inclusion of swine liver protein hydrolysate. Means followed by distinct letters in the columns indicate significant differences by the Tukey’s test (*p* < 0.05).

## Supplementary Materials

The following are available online at https://www.mdpi.com/2076-2615/9/6/311/s1, Figure S1: The OPA-reaction; Table S1: Value of constants a, b, and h_tot_ for different protein raw materials.
