# Supplementary material for: Palatability of Protein Hydrolysates from Industrial Byproducts for Nile Tilapia Juveniles
Source: Animals (Basel). 2019 May 31;9(6):311. doi: 10.3390/ani9060311 (PMC6616487; doi:10.3390/ani9060311)
Supplement: Supplementary file 1 [file animals-09-00311-s001.pdf]

According to Nielsen et al (2001), in protein hydrolysis, the key parameter for monitoring the reaction is the degree of hydrolysis (DH). DH is defined as the percentage of cleaved peptide bonds:

DH = h/htot * 100%

where **htot is the total number of peptide bonds per protein equivalent,** and **h is the number of hydrolyzed bonds. htot is dependent on the amino-acid composition of the raw material.**

Several methods of monitoring the DH during protein hydrolysis have been described in the literature.

Nielsen et al (2001) developed a new method to monitoring the degree of hydrolysis (DH).

To provide a basis for developing a suitable method, a reaction was selected between amino groups and o-phthaldialdehyde (OPA) in the presence of beta-) mercaptoethanol forming a colored compound detectable at 340 nm in a spectrophotometer (Figure 1).

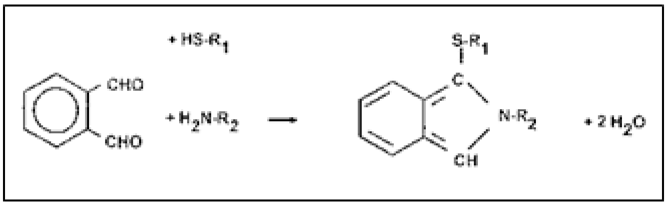

Figure 1—The OPA-reaction. OPA reacts with primary amino groups and a SH-compound (dithiothreitol, DTT) to form a compound that will absorb light at 340 nm.

Calculation

Determination of h:

Serine-NH2 = ODsample - ODblank/ODstandard - ODblank* 0.9516 meqv/L *0.1 * 100/ X * P

Serine-NH2 = (serine as the standard, since in reactions serine shows a response very close to the average response of amino acids)

where serine-NH2 = meqv serine-NH2/g protein;

X = g sample;

P = protein % in sample;

0.1 is the sample volume in liter (L).

h is then:

h = (serine-NH2 - b)/a meqv/g protein

where **a** and **b** are shown in **Table 1** for specific raw materials.

Table 1—Value of constants a, b, and h_{tot} for different protein raw materials (Adler-Nissen 1986)

Protein	a	b	h_{tot}
Soy	0.970	0.342	7.8
Whey*	1.00	0.40	8.8
Meat*	1.00	0.40	7.6
**Fish***	**1.00**	**0.40**	**8.6** (these are the values to use in fish hydrolysates)

*** When raw material has not been examined, then a and b are estimated to be 1.00 and 0.40, respectively.**

Calculation of DH:

DH = h/htot * 100 %

where **htot** for specific raw materials is found in **Table 1**.

The conclusions of the work of Nielsen et al (2001):

The new method has proved to be more accurate than the previous method based on TNBS reaction. Furthermore, the OPA method can be used to follow the hydrolysis reaction during hydrolysis and is much less time-consuming than the TNBS method. Because results are available 2 min after the sample is taken, the OPA method can be used in production to monitor DH during hydrolysis. A final benefit is that the OPA reagent is more stable and less toxic than the TNBS reagent. It is therefore suggested that the OPA method is used for determining DH in hydrolyzed proteins, in food products, as well as feed products.
